# δ^13^C of terrestrial vegetation records Toarcian CO_2_ and climate gradients

**DOI:** 10.1038/s41598-019-56710-6

**Published:** 2020-01-10

**Authors:** Wolfgang Ruebsam, Matías Reolid, Lorenz Schwark

**Affiliations:** 1 Department of Organic and Isotope Geochemistry, Institute of Geoscience, University of Kiel, Kiel, Germany; 2 Departamento de Geología and CEACT, Universidad de Jaén, Jaén, Spain; 3 WA-OIGC, Curtin University, Perth, Australia

**Keywords:** Climate sciences, Climate change, Palaeoclimate

## Abstract

Throughout Earth’s history, variations in atmospheric CO_2_ concentration modulated climate. Understanding changes in atmospheric carbon cycle is therefore pivotal in predicting consequences of recent global warming. Here, we report stable carbon isotopes (δ^13^C) of molecular land plant fossils complemented by bulk organic and inorganic carbon fractions for early Toarcian (Early Jurassic) sediments that coincided with global warming and a carbon cycle perturbation. The carbon cycle perturbation is expressed by a negative excursion in the δ^13^C records established for the different substrates. Based on differences in the magnitude of the carbon isotope excursion recorded in land plants and marine substrates we infer that the early Toarcian warming was paralleled by an increase in atmospheric CO_2_ levels from ~500 ppmv to ~1000 ppmv. Our data suggest that rising atmospheric CO_2_ levels resulted from the injection of  ^12^C-enriched methane and its subsequent oxidation to CO_2_. Based on the cyclic nature of the CIE we concluded that methane was released from climate sensitive reservoirs, in particular permafrost areas. Moderate volcanic CO_2_ emissions led to a destabilization of the labile permafrost carbon pool triggering the onset of Toarcian climate change only. The main carbon cycle perturbation then subsequently was driven by a self-sustained demise of a carbon-rich cryosphere progressing from mid to high latitudes as reflected by latitudinal climate gradients recorded in land plant carbon isotopes.

## Introduction

Anthropogenic fossil carbon emissions steadily increase atmospheric CO_2_ levels and thereby impact on Earth’s climate and carbon cycle^1^. As a consequence rising global temperatures can led to a reactivation of carbon stored in permafrost regions that upon its release to the atmosphere will further accelerate global warming^2^. Melting polar ice caps and sea level rise, climate extremes and enhanced stress for marine and continental ecosystems have been proven to be direct consequences of global warming^3–5^. Predictions on the evolution of Earth’s climate system, the carbon cycle and the response of ecosystems are, however, problematic. Thus, investigation of sediment archives that record ancient climate perturbation can serve as analogues for recent climate change and can thereby guide in predicting consequences of global warming and its cascade of consequences.

Here, we address changes in Earth’s climate and carbon cycle that occurred in conjunction with the early Toarcian Oceanic Anoxic Event (Early Jurassic; ∼183 Ma). This study utilizes stable carbon isotopes recorded in different substrates, facilitating the reconstruction of changes in the global carbon cycle, atmospheric CO_2_ levels and latitudinal climate gradients during the early Toarcian global warming.

## Background

Around the globe, sediment archives that span the early Toarcian record profound environmental changes. A rapid high-amplitude sea level rise paralleled by a decline in oxygen isotope values of macrofossil calcite, has been interpreted to reflect a rise in sea water temperatures that was potentially accompanied by a reduction in the volume of land-based ice caps^6–9^. Rising global temperatures evolved parallel to an increase in atmospheric CO_2_ level inferred from stomata data^10^. In the marine realm global warming led to expansion of marine death zones and triggered the genesis of the Toarcian Oceanic Anoxic Event (T-OAE)^11^, whereas on land it caused substantial shifts in floral assemblages^10,12,13^.

A hallmark of the early Toarcian is a negative carbon isotope excursion (CIE) that is interpreted to reflect a global carbon cycle perturbation, caused by injections of ^12^C-enriched carbon into Earth’s hydro-atmosphere system^9,14–18^. Carbon sources are debated controversially and comprise a volcanic CO_2_ and/or thermogenic CH_4_ associated with the emplacement of the Karoo-Ferrar Large Igneous Province of southern Gondwana^10,19^, destabilization of methane hydrates^14,16^, increased rates of wetland methanogenesis^17^, or permafrost decay and thermokarst blowout events during global warming^9^. The CIE has been reported in marine and terrestrial organic matter as well as in marine carbonates^10,12,14,15^, suggesting that the carbon cycle perturbation affected the entire exchangeable carbon reservoir. A decline in δ^13^C documented in land plant-derived lipids indicates atmospheric ^13^C depletion and substantiates a perturbation of the atmospheric carbon cycle^18,20,21^. However, current δ^13^C records of land plant-derived lipids cover only a brief stratigraphic interval and provide no information on the recovery phase of the CIE and on the long-term evolution of the atmospheric carbon reservoir. Moreover, information on atmospheric CO_2_ concentration and its absolute change during the early Toarcian warming event are based on stomata data from a single section only and span the onset of the CIE^10^. Reconstruction of atmospheric CO_2_ concentration may further be complicated by stratigraphic gaps and methodological limitation^10,22^.

Here we utilize compound-specific carbon isotope data of land plant wax lipids to reconstruct changes in the atmospheric carbon reservoir across the early Toarcian carbon cycle perturbation and the associated climate event. The δ^13^C analysis of land plant-derived wax lipids, compounds not affected by the differential preservation of fossilized wood fragments^15^, provide a robust method for reconstructing changes in the isotopic composition of the atmospheric carbon reservoir. The compound-specific δ^13^C record is complemented by δ^13^C data from marine calcite that reflect changes in the oceanic carbon reservoir. The reconciliation of δ^13^C excursions in land plant and marine substrates allows reconstruction of changes in the entire exchangeable carbon reservoir. Moreover, parallel evaluation of marine and terrestrial carbon isotope excursions provide information not only on changes in atmospheric CO_2_ concentration but also on absolute atmospheric CO_2_ levels prior to and during the early Toarcian carbon cycle perturbation^23,24^.

### Study site

**Figure 1:**
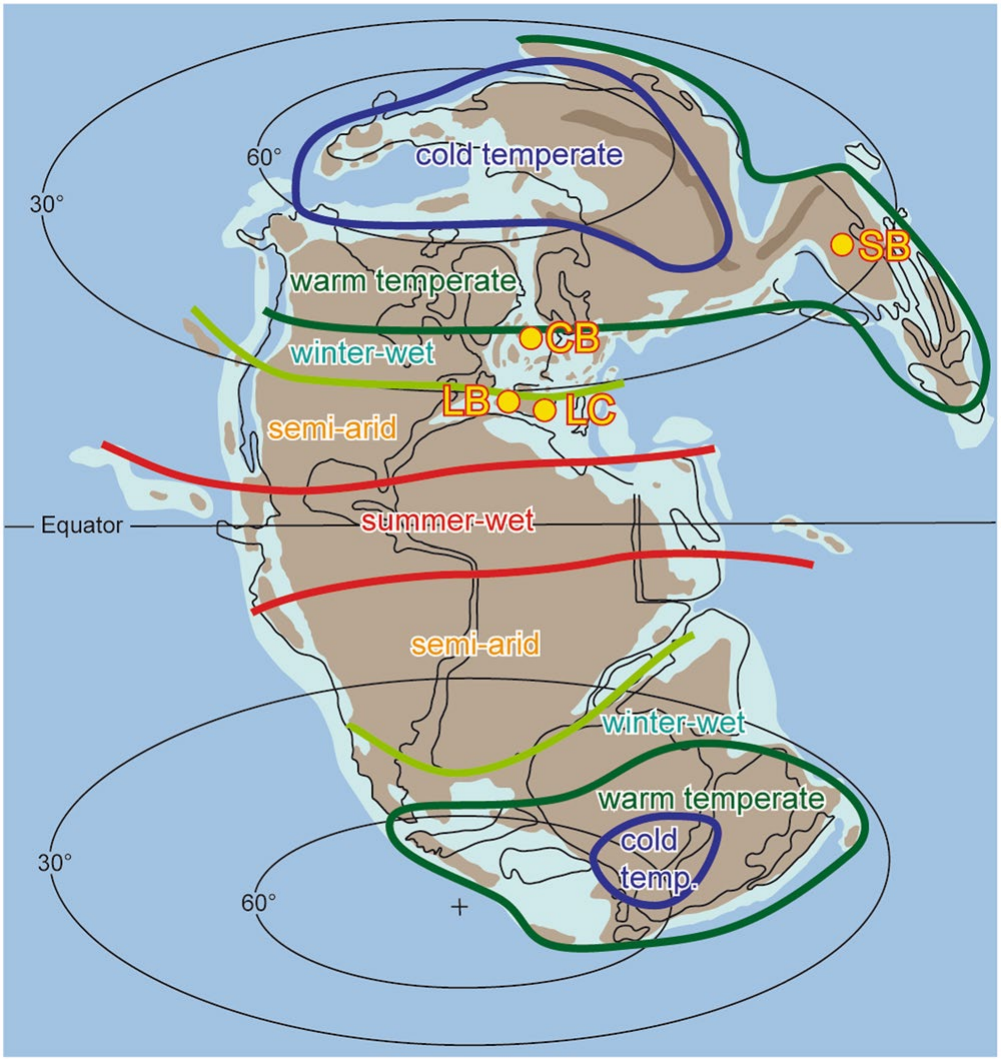
Earth’s paleogeography and distribution of climate belts during the Early Jurassic (modified after Rees (ref. ^27^)). Paleogeographic map generated with Adobe Illustrator CC 2019, http://www.adobe.com/products/illustrator.html. Locations mentioned in the text are indicated (CB: Cleveland Basin, UK; LB: Lusitanian Basin, Portugal; LC: La Cerradura, Iberian Basin, Spain; SB: Sichuan Basin, China).

In this study we investigated upper Pliensbachian to lower Toarcian sediments, represented by the *Emaciatum* to *Serpentinum* ammonite zones and the NJT5b to NJT6 nannofossil zones, cropping out at La Cerradura (Subbetic, southern Spain)^25^. Ammonite assemblages in combination with coccolithophore-based biostratigraphic data indicate that the sediments can be correlated with the T-OAE^26^, which is further supported by paleontological and geochemical data^25^. Sediments, mainly marlstone-limestone alternation, were deposited in a fragmented marine platform with hemipelagic sedimentation at a paleolatitude of about 26°N at the southern Iberian paleomargin. Floral assemblages suggest that during the Early Jurassic (183 Ma) the study site was located in the semi-arid climate belt^27^ (Fig. 1).

## Results and Discussion

### An atmospheric record of the toarcian carbon cycle perturbation

**Figure 2:**
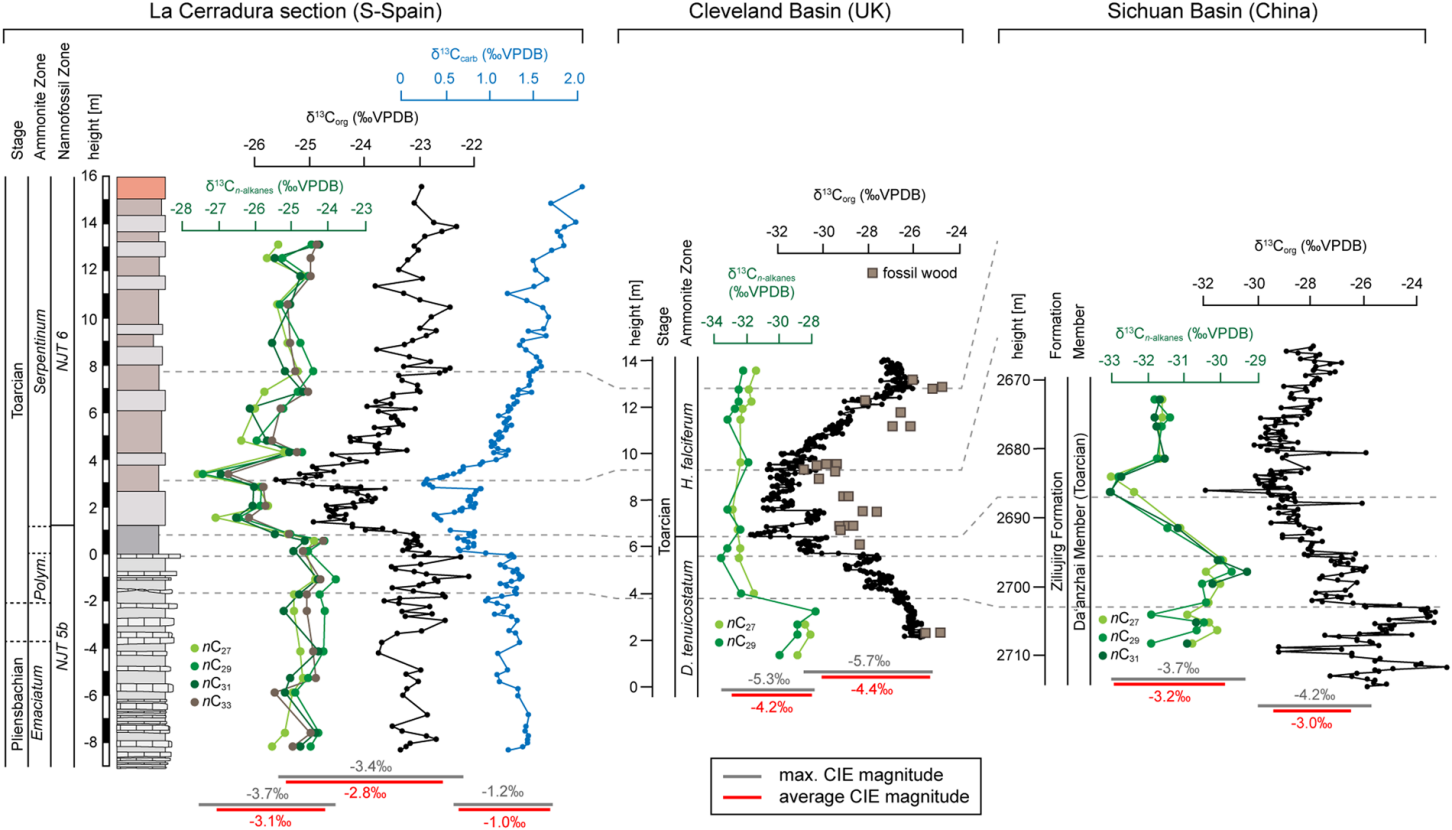
Stable carbon isotopes determined on fossilized land plant lipids, bulk organic and carbonate carbon from the La Cerradura section (southern Spain) show a stepped negative CIE at the *Polymorphum-Serpentinum* zonal transition, confirming that the early Toarcian carbon cycle perturbation affected the entire exchangeable carbon reservoir. At La Cerradura terrigenic lipids record a magnitude in Δ^13^C_*n*-alkane_ of −3.7‰, which is comparable to the magnitude documented for the CIE recorded in plant wax alkanes from the Cleveland (UK)^20^ and Sichuan Basins (China)^18^. Differences in the absolute values of plant wax alkane δ^13^C reflect environmental conditions in different climate belts (see Fig. 1).

The early Toarcian carbon cycle perturbation is expressed in δ^13^C_org_ and δ^13^C_carb_ data by negative excursions of −3.4‰ and −1.2‰, respectively (Table 1 in the SI). A shift towards lower δ^13^C values occurred in a stepwise manner at the *Polymorphum-Serpentinum* zonal transition (Fig. 2). Stratigraphic position as well as pattern and pacing of the CIE at La Cerradura match trends from other locations documenting a multiphasic carbon cycle perturbation^9,15,16^.

The δ^13^C signatures of terrestrial *n*-alkanes recording a negative CIE with a magnitude of −3.7‰ (−3.1‰ on average) (Fig. 2) parallel the high-resolution δ^13^C bulk data (Table 2 in the SI). The stepped CIE character is documented in the δ^13^C_*n*-alkane_ record, confirming that the CIE reflects multiple re-occurring carbon injections into the Earth’s ocean-atmosphere system^9,16^. Moreover our data unequivocally demonstrates that the Toarcian carbon cycle perturbation affected not only the marine but also the atmospheric carbon reservoir, as previously shown by Pienkowski *et al*. (ref. ^12^) and Hesselbo *et al*. (refs. ^14,15^). The −3.7‰ magnitude of the CIE at La Cerradura is similar to that reported in long chain *n*-alkanes from the Sichuan Basin (China)^18^, but is slightly lower than the −5.3‰ CIE (−4.2‰ on average) determined for terrigenic *n*-alkanes from the Cleveland Basin (UK)^20^ (Fig. 2). Differences in the magnitude may originate from low stratigraphic coverage of compound-specific δ^13^C values and/or stratigraphically incompleteness of the sections. Higher and variant magnitudes in the range from −3.5 to −8.0‰ reported in δ^13^C_wood_ (Table 3 in the SI)^12,14,15,21^ can be attributed to differential preservation states (e.g. jet, charcoal), molecular heterogeneity, or taxonomic impact on the isotopic signature of fossil wood^15^. Moreover, when preserved as jet (degraded wood), microbial reworking and impregnation by marine taxa during exposure to seawater, can alter the initial δ^13^C signature^15^.

While δ^13^C_*n*-alkane_ records for different basins show similar trends and magnitudes of the CIE, their absolute δ^13^C values differ. The δ^13^C_*n*-alkane_ records from the Cleveland and Sichuan basins both show base values more depleted in ^13^C by about 4 to 5‰ when compared with base values from La Cerradura (Fig. 2). This offset relates to latitudinal climate gradients associated with different floral assemblages and precipitation rates impacting on δ^13^C of land plants^23,28–30^ (Fig. S4 in the SI). During the Early Jurassic the Cleveland and Sichuan basins were located in a winter-wet temperate climate belt, while southern Iberia was situated in the winter-wet to semi-arid climate belt^27^ (Fig. 1). Lower precipitation rates in the latter are expressed in a dominance of xerophytic flora^31,32^ and are evident in clay mineral assemblages^33^. Accordingly, differences in the δ^13^C_*n*-alkane_ values from the different basins reflect a strong latitudinal climate gradient. A dominance of exceptionally long *n*-alkanes in samples from La Cerradura (Figs. S1, S2 in the SI) confirms organic matter contributions from xerophytic flora. Therefore, δ^13^C_*n*-alkane_ at La Cerradura records the terrestrial δ^13^C pool as part of the global carbon cycle.

### Quantifying atmospheric CO_2_ levels across the early Toarcian CIE

The early Toarcian CIE was associated not only with changes in the isotopic composition of the exchangeable C-reservoir, but also with changes in atmospheric *p*CO_2_ levels. Based on stomata data McElwain *et al*. (ref. ^10^) reported *p*CO_2_ values in the range 350 to 1200 ppmv and 250 to 1800 ppmv in pre-CIE and CIE intervals, respectively. However, fragmentary deposition, stratigraphic incompleteness, and very low number of data points complicate robust stomatal *p*CO_2_ estimates. Moreover, there is also a poor calibration of the stomata proxy that can also respond towards environmental factors other than atmospheric CO_2_^22,34^.

An alternative approach for determining ancient *p*CO_2_ levels is based on the observation that the isotopic fractionation of C3 land plants will vary not only with precipitation rates, but also with *p*CO_2_^23,24,35^. This CO_2_ effect results in a higher isotopic fractionation when *p*CO_2_ levels increase and thereby cause higher CIE magnitudes in terrigenic than in marine substrates^24^. Offsets in CIE magnitude of terrigenic versus marine substrates thus facilitate determination of absolute atmospheric *p*CO_2_ levels^24,35^ (for details we refer to the supplementary information). However, as pointed out by Schubert & Jahren (ref. ^23^) and Lomax *et al*. (ref. ^36^) under enhanced water stress the carbon isotopic signatures of C3 plants vary as a function of precipitation rates and then do not unambiguously reflect past atmospheric CO_2_ concentration. According to recent observations, a strong impact of precipitation rates on δ^13^C of land plant biomass has been documented for vegetation in areas with mean annual precipitation rates < 2200 mm/year. On the contrary, precipitation seems to have no significant impact on the land plant δ^13^C in areas with high mean annual precipitation rates^23^. The dominance of xerophytic flora in the southern Iberian paleomargin, which here is represented by the La Cerradura section, suggests low paleo-precipitation rates and eventually enhanced paleo-water stress^31,32^. When compared to localities at higher latitudes, lower paleo-precipitation rates also manifested themselves in the ^13^C-enrichment of the land plant biomass. We can, however, speculate only about absolute paleo-precipitation rates at the southern Iberian paleomargin, which complicate evaluating the impact of water stress on the δ^13^C land plant biomass.

In order to calculate *p*CO_2_ levels and to minimize the effect of different paleo-precipitation rates, we compared data from the La Cerradura section, located in a semi-arid climate belt, with data from Yorkshire (UK)^20^ and from China^18^ that were both situated in a humid climate belt (Fig. 1). In particular the δ^13^C_*n*-alkane_ data from sites situated in a humid climate are supposed to vary in dependency of changing atmospheric CO_2_ levels^36^. Moreover, a CO_2_ dependence of the land plant δ^13^C has also been documented for vegetation growing under low water treatment^29^. It is therefore reasonable to assume that changes in δ^13^C _*n*-alkane_ at all sites will also vary as a function of changes in the atmospheric CO_2_ concentration. This assumption is underpinned by the consistent evolution and similar magnitudes of the CIE seen in the δ^13^C _*n*-alkane_ at all sites investigated (Fig. 2).

Based on δ^13^C_*n*-alkane_ data from La Cerradura (this study), Yorkshire^20^ and China^18^ we calculated a maximal magnitude in the CIE_terrigenic_ of −4.2‰ (−3.1‰ on average). A higher CIE_terrigenic_ of −5.4‰ is achieved when including δ^13^C data of fossil wood and phytoclasts (Table 3 in the SI). Following the approach of Schubert & Jahren (ref. ^24^), we determined the magnitude of the CIE in marine substrates (CIE_marine_) by using δ^13^C_carb_ data from oxygenated marine basins only. This includes data from organic matter-lean sediments deposited at the southern part of the West Tethys Shelf. At these areas the seafloor preferentially remained oxygenated throughout the early Toarcian^37^. For such settings organic matter-induced carbonate diagenesis and/or CO_2_ recycling in stratified water bodies that may alter the δ^13^C signature can assumed to be minimal or can even be excluded^38,39^. Carbon isotope data from marine organic matter is not included in our calculation, as δ^13^C_org_ values can be affected by mixing of organic matter of marine phototrophic and non-phototrophic organisms or land plants^24^. We  calculated an average CIE_marine_ of −2‰ (Table 3 in the SI), which is similar to the −2 to −3‰ estimate by Suan *et al*. (ref. ^40^). Using the δ^13^C_*n*-alkane_ based CIE_terrigenic_ and the CIE_marine_ we calculated a ΔCIE (ΔCIE = CIE_terrigenic_ – CIE_marine_) of −1.5 and −2.2‰, for average and maximal values of the CIE_terrigenic_, respectively. Including δ^13^C data from fossil wood yields a ΔCIE of about −3.4‰.

Calculation of *p*CO_2_ levels prior to the CIE (*p*CO_2(init)_) and during the climax of the CIE (*p*CO_2(CIE)_) further requires an estimation for the Δ*p*CO_2_ that here is derived from mass balance calculations in dependency of the CIE_marine_ and the isotopic signature of the respective carbon source. We calculated Δ*p*CO_2_ values for carbon sources with isotopic signatures characteristic for: i) biogenic methane emissions (δ^13^C: −70‰^41,42^) ii) gas hydrates (δ^13^C: −60‰^43^), iii) thermogenic methane (δ^13^C: −35‰^43^) and iv) a source dominated by volcanogenic CO_2_ (δ^13^C: > −10‰^42^) (for details see supplementary information).

**Figure 3:**
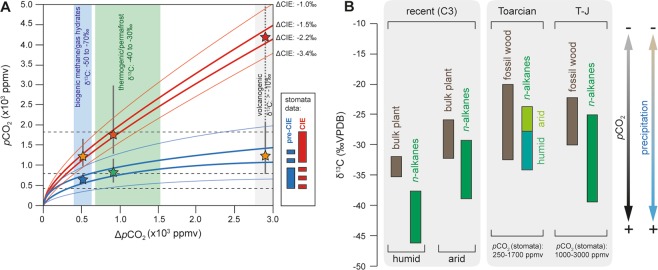
(**A**) Reconstruction of atmospheric *p*CO_2_ prior to and during the early Toarcian CIE. In dependency of the carbon source and its isotopic signature different and partly contrasting CO_2_ scenarios can be proposed. The best fit scenario is achieved for carbon sources enriched in ^12^C, suggesting that CIE and climate change were driven by carbon injections from cryosphere collapse^9^, or gas hydrates and wetlands^17^ (low *p*CO_2_ scenario, blue and orange asterisks). Such a scenario agrees with stomata-based *p*CO_2_ estimates^10^. Contribution from thermogenic methane released from fossil hydrocarbon sources would be plausible as well (moderate *p*CO_2_ scenario, green and red  asterisks). On the contrary, scenarios invoking volcanic CO_2_ emissions as primary driver of the early Toarcian carbon cycle perturbation are not supported by our data (high *p*CO_2_ scenario, orange and red asterisks). Vertical grey bars indicate uncertainties of *p*CO_2_ determinations. (**B**) Impact of *p*CO_2_ levels and precipitation rates on the δ^13^C of land plant biomass (T-J: Triassic-Jurassic boundary; see supplementary information for additional information and references). Latitudinal climate and vegetation gradients cause offsets in δ^13^C absolute values.

For an isotopically-light carbon source (−70 to −50‰) and a ΔCIE of −2.2‰ and −3.4‰, we calculated values for *p*CO_2(init)_ ∼600 ppmv and of ∼400 ppmv, respectively, whereas for *p*CO_2(CIE)_ we obtained 1200 and 850 ppmv, respectively (Fig. 3). Initially low pre-CIE CO_2_ estimates will be affected by a maximum uncertainty of about +350/−100 ppmv, while a higher maximum uncertainty of about +1000/−400 ppmv must be assumed for CO_2_ estimates during the CIE^44^. Errors result from uncertainties in the model-curve fit of the experimental data^23^ and from uncertainties in the input parameters used to calculate *p*CO_2_^44^ (Fig. S3 in the SI). The error range also includes uncertainties arising from unknown paleoenvironmental conditions under which fossil plants grew^44^. The uncertainty can be assumed to be comparable to those associated with other methods for past *p*CO_2_ reconstruction^22,44^. Isotope-based estimates are close to the stomata-based *p*CO_2_ assessment^10^. However, in contrast to McElwain *et al*. (ref. ^10^), our data attest to a doubling in *p*CO_2_ instead of a threefold increase (Fig. 3). Our results strongly suggest that an early Toarcian carbon cycle perturbation was caused by carbon released in form of ^12^C-enriched methane from a cryosphere collapse^9^ or, alternatively, from marine gas hydrates^14^ or wetlands^17^.

With respect to uncertainties in the ΔCIE value and in the δ^13^C-based CO_2_ reconstruction^44^, thermogenic methane release from thermal alteration of organic matter-rich sediments during the Karoo-Ferrar emplacement^10,19^ would be plausible as well. Such a scenario is, however, not supported by geochemical data^45,46^ and is further difficult to reconcile with the orbitally-forced cyclic pattern of the CIE that is only explained by carbon release from climate-sensitive reservoirs responding to changes in Earth’s solar orbit^9,16^. On the contrary, release of biogenic and thermogenic methane from glacier- and permafrost-capped reservoirs would be a plausible scenario^9^ that is supported by recent observations^41^.

Assuming volcanic CO_2_ emission as being the major driver of the early Toarcian climate change would require the release of enormous amounts of CO_2_ that would have shifted *p*CO_2_ levels from about 1000 ppmv during pre-event times to more than 4000 ppmv during the CIE (Fig. 3). Thus, direct volcanic CO_2_ emissions fail in explaining both, the magnitude of the CIE and of climate change (Fig. 3). A plausible scenario would be that the emplacement of the Karoo-Ferrar Large Igneous province released small quantities of volcanic CO_2_ and eventually some thermogenic methane from Gondwana coals. Both initiated a moderate rise in global temperatures, triggering the release of ^12^C-enriched carbon from mid-latitudinal climate-sensitive reservoirs. In combination with changes in Earth’s solar orbit this atmospheric carbon increase stimulated a self-sustaining cryosphere demise prograding to higher latitudes and thereby releasing even more cryosphere-stored carbon, a process assumed to be the major driver of the early Toarcian climate and environmental change^9^. Our results allow us to postulate that the early Toarcian carbon cycle perturbation and associated climate changes were driven primarily by the release of huge quantities of ^12^C-enriched methane from climate sensitive cryosphere reservoirs.

## Conclusions

The compound-specific carbon isotope record for land plant-derived long-chain *n*-alkanes from Iberia provides a robust long-term record of changes in the atmospheric carbon reservoir that occurred in concert with the early Toarcian global warming. The presence of a negative CIE in long-chain *n*-alkanes that parallels bulk organic and inorganic δ^13^C trends confirms ^13^C-depletion of the entire exchangeable carbon reservoir, in particular atmospheric ^13^C-depletion. Based on offsets in the magnitude of the CIE reported in terrigenic and marine substrates, we calculated a doubling in atmospheric CO_2_ levels paralleled the carbon cycle perturbation and global warming. Carbon added to the ocean-atmosphere system was strongly enriched in ^12^C derived from climate-sensitive cryosphere reservoirs. Karoo-Ferrar volcanism may have triggered global warming but volcanic CO_2_ emissions fail to explain the magnitude of the carbon cycle perturbation. Accordingly, volcanic CO_2_ was only a trigger but not the driver of the early Toarcian climate change, which was caused by successive and self-attenuating cryosphere collapse. Our data suggest that environmental changes that occurred concomitant to the T-CIE were linked to the release of huge amount of cryosphere methane to the Earth’s ocean-atmosphere system.

## Material and Methods

### Sampling

Geochemical analysis have been performed at sample material that has been taken at the La Cerradura section after removal of surface rocks that potentially experience alteration due to weathering. All samples have been taken at least 30 cm below surface. Rock samples were crushed and powdered in order to obtain a homogenous and representative sample. Prior to geochemical analysis the powdered sample material was dried in an oven at 40 °C for 48 h.

### Stable carbon isotope analysis of the bulk organic matter and carbonate

Stable carbon isotope analysis for bulk organic carbon (δ^13^C_org_) were performed on decalcified sample material^9^. Decalcification was achieved by treating the sample material with hydrochloric acid (HCl, 10% and 25%) to remove carbonate-bound and if present dolomite-bound carbon. Afterwards, samples were washed, neutralized with deionized water and dried in an oven at 40 °C for 48 h. Stable carbon isotope analysis was performed using a Thermo Finnigan Delta V isotope ratio mass spectrometer coupled to a Flash EA via a Conflow III interface.

The carbonate fraction was measured for its carbon isotopes using a Kiel III carbonate preparation line connected to a Thermo Fisher 252 mass spectrometer. Powered and homogenized samples were treated with 103% phosphoric acid at 70 °C^47^. Carbon isotope ratios of the organic matter and the carbonate are expressed in conventional delta notation: δ_sample_ (‰) = [(R_sample_ − R_standard_)/R_standard_ − 1] × 1000, where R is the ratio of ^13^C/^12^C of the sample and the V-PDB standard for carbon. Reproducibility and accuracy were monitored by replicate standard and sample analysis and are better than 0.1‰.

### Stable carbon isotope analysis of land plant *n*-alkanes

Total lipid extracts for selected samples were obtained from solvent extraction using a Soxhlet apparatus. As extraction solvent we used a mixture of dichloromethane (DCM) and methanol (MeOH) (9:1, v/v). Similar to the method applied by Ruebsam *et al*. (ref. ^48^) total bitumen extracts were separated into aliphatic, aromatic and polar hydrocarbon fractions by silica gel-column chromatography (8 ml SPE column, 2.8 g Silica 60 mesh, 25–40 μm) using solvents with increasing polarity in an LCTECH automated SPE system. The aliphatic hydrocarbon fractions were treated with activated copper turnings in order to remove elemental sulfur. GC–MS measurements of the aliphatic hydrocarbon fractions were performed on an Agilent 5975B MSD interfaced to an Agilent 7890 A GC equipped with a quartz capillary (Agilent DB1-HT; 60 m length, 0.25 mm inner diameter, 0.25 μm film thickness). The temperature program of the GC oven used was: 70 °C (5 min isothermal) to 140 °C at 10 °C/min, then to 325 °C at 3 °C/min (held for 7 min). The quadrupole MS was operating in scan mode in the m/z 50 to 750 range. Compounds of interest were identified via characteristic mass spectra and were integrated manually using the GC/MSD Masshunter Software (Agilent Technologies)^48^.

Aliphatic hydrocarbon fractions of all samples analyzed are clearly dominated by odd-numbered long-chain *n*-alkanes (Fig. S1 in the SI), originated in land plants^49^. Cyclic aliphatic hydrocarbons (steroids, hopanoids) are present as well, but occur at very low abundances (acyclic/cyclic > 10; Figs. S1 and S2 in the SI). Moreover, the temperature program of the GC oven was modified to minimize co-elution of the odd-numbered *n*-alkanes with cyclic aliphatic hydrocarbons (Fig. S2 in the SI). Due to the clear dominance of long-chain *n*-alkanes and the absence of co-elution with cyclic aliphatic hydrocarbons compound-specific δ^13^C analysis for the long-chain *n*-alkanes was performed on untreated aliphatic hydrocarbon fractions, without previous mole-sieving as commonly applied^50^.

Gas chromatography–isotope ratio mass spectrometry (GC–irMS) was performed following the methodology described in Plet *et al*. (ref. ^50^) using a Thermo Scientific Trace GC Ultra interfaced to a Thermo Scientific Delta V Advantage mass spectrometer via a GC isolink and a Conflow IV. The δ^13^C values of the compounds were determined by integrating the ion currents of masses 44, 45 and 46, and are reported in permil (‰) relative to the VPDB standard. Reported values are the average of at least two analyses with standard deviation of <0.5‰.

### Calculation of *p*CO_2_ levels

Calculation of *p*CO_2_ levels follows the approach by Schubert & Jahren (ref. ^24^) and is based on the differences in the magnitude of a CIE reported in land plant organic matter and marine substrates. Assessment of methodical uncertainties is based on the work by Cui and Schubert (ref. ^44^) and varies as a function of absolute *p*CO_2_ concentration. Details on the calculations are provided in the supplementary information.

## Supplementary information

Supplementary Information.

Supplementary Information2.

Supplementary Information3.

Supplementary Information4.
