# Supplementary material for: δ13C of terrestrial vegetation records Toarcian CO2 and climate gradients
Source: Sci Rep. 2020 Jan 10;10:117. doi: 10.1038/s41598-019-56710-6 (PMC6954244; doi:10.1038/s41598-019-56710-6)
Supplement: Supplementary file 1 — Supplementary Information. [file 41598_2019_56710_MOESM1_ESM.pdf]

Supplementary information

δ^{13}C of terrestrial vegetation records Toarcian CO_2 and climate gradients

Wolfgang Ruebsam[1], Matías Reolid[2], and Lorenz Schwark[1,3]

[1] Department of Organic and Isotope Geochemistry, Institute of Geoscience, University of Kiel, Germany, e-mail: wolfgang.ruebsam@ifg.uni-kiel.de

[2] Departamento de Geología and CEACT, Universidad de Jaén, Jaén, Spain

[3] WA-OIGC, Curtin University, Perth, Australia

Compound-specific carbon isotope analysis of long-chain *n*-alkanes

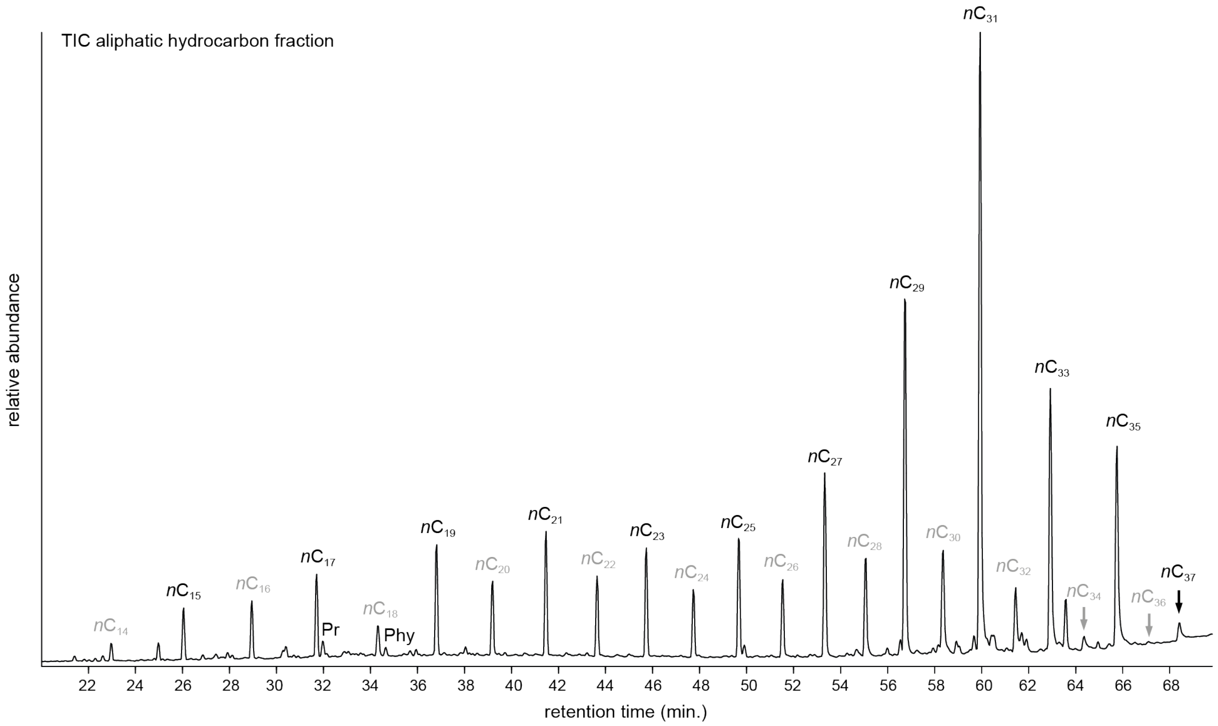

Fig. S1. Representative GC-MS trace of a total ion chromatogram (TIC) of an aliphatic hydrocarbon fraction. Note the clear dominance of *n*-alkanes in the aliphatic hydrocarbon fraction.

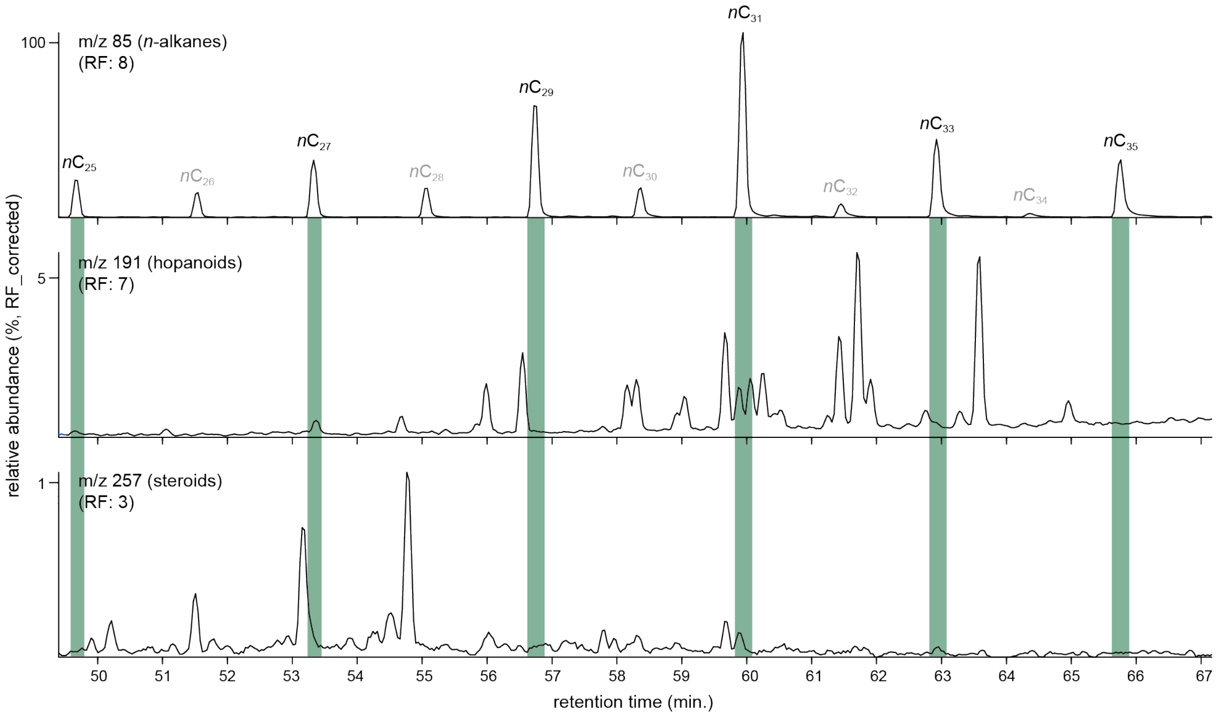

Fig. S2. Representative m/z 85, m/z 191 and m/z 257 traces for long-chain *n*-alkanes, hopanes and dia-sterenes, respectively. Relative abundances of the single compound classes are given in percentage corrected for the individual response factors. Co-elution only occurs for the *n*-alkane *n*C_{31} that elutes between hopanoids. However, due to the clear dominance of the n-alkanes over the cyclic compounds, a strong effect on the *n*-alkane $\delta^{13}C$ can be excluded.

Calculation on pCO_2 levels

Calculation of pCO_2 levels prior to the CIE ($pCO_{2(init)}$) and during the climax of the CIE ($pCO_{2(CIE)}$) follows the approach by Schubert & Jahren (ref.[1]) that is based on the observation that the $\delta^{13}C$ of C3 plants shows a hyperbolic relationship with atmospheric CO_2 levels (CO_2 effect). This relation is expressed by the equation 1:

$$\Delta\delta^{13}C = \frac{(A)(B)(pCO_2+C)}{A+(B)(pCO_2+C)} \quad (1)$$

where A, B and C are constants derived from experimental data (A = 28.21; B = 0.21; C = 25)[1].

The CO_2 effect results in a larger magnitude of a CIE recorded in terrestrial substrates (land plants) ($CIE_{terrestrial}$) versus marine substrates (CIE_{marine}) ($\Delta CIE = CIE_{terrestrial} - CIE_{marine}$). The ΔCIE is expressed by the equation 2:

$$\Delta CIE = \frac{(A)(B)(pCO_{2(init)}+C)}{A+(B)(pCO_{2(init)}+C)} - \frac{(A)(B)(pCO_{2(CIE)}+C)}{A+(B)(pCO_{2(CIE)}+C)} \quad (2)$$

Where, $pCO_{2(init)}$ and $pCO_{2(CIE)}$ are the atmospheric CO_2 levels immediately before the CIE and at the height of the CIE, respectively. Knowledge of ΔCIE can then be used to calculate $pCO_{2(init)}$ and $pCO_{2(CIE)}$, but requires an estimate for the change in pCO_2 (ΔpCO_2):

$$\Delta pCO_2 = pCO_{2(CIE)} - pCO_{2(init)} \quad (3)$$

Estimates for ΔpCO_2 can be calculated from mass balance equations (equation 4) and will depend on the $\delta^{13}C$ values of the source[2]:

$$\Delta pCO_2 = \frac{-(CIE_{marine})(M_{init})(0.3)}{\delta^{13}C_{init} - \delta^{13}C_{source}} \quad (4)$$

where CIE_{marine} is the magnitude of the CIE recorded in marine substrates (here carbonates), M_{init} is an estimate for the initial size of the exchangeable C-reservoir (here approx. 45000 Gt[2,3]), $\delta^{13}C_{init}$ is the initial isotopic signature of the exchangeable

reservoir (+2‰)[2,3], $\delta^{13}C_{final}$ is the $\delta^{13}C$ value at the CIE ($\delta^{13}C_{final} = \delta^{13}C_{initial} + CIE_{marine}$) and $\delta^{13}C_{source}$ is the $\delta^{13}C$ value of the source causing the CIE. Estimates for ΔpCO_2 were calculated for i) strongly ^{12}C-enriched C-sources (methane emissions from thawing permafrost and wetlands: $\delta^{13}C$ = -70‰[4,5] and marine gas hydrates: $\delta^{13}C$ = -60‰[2], ii) a C-source moderately enriched in ^{12}C (e.g. thermogenic methane: $\delta^{13}C$ = -35‰[6]) and iii) a C-source not significantly enriched in ^{12}C (volcanic CO_2 emission: $\delta^{13}C$ > -10‰[2]). Note: thawing permafrost here refers to methane emissions from reservoirs trapped within and below by permafrost. The $\delta^{13}C$ value of CO_2 originating from permafrost decomposition is about -30‰[1]. An addition of 1 Gt C will result in an increase in pCO_2 by 0.3 ppmv, which is indicated by the constant of 0.3[1].

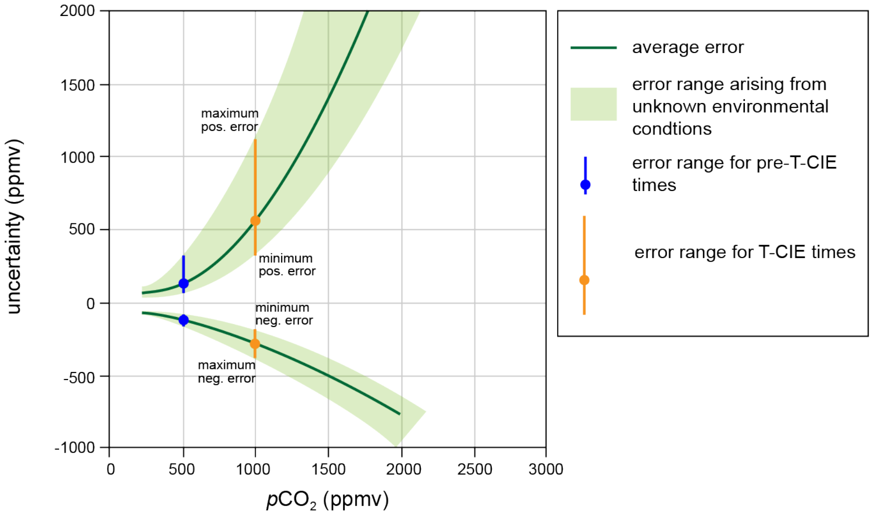

Fig. S3. Positive and negative errors of pCO_2 reconstructions that are based on changes in the $\delta^{13}C$ of land palnts[1]. The green shaded area reflects uncertainties resulting from unknown paleoenvironmental conditions under which plants grew. For details in the assessment on methodological uncertainties we refer to Cui and Schubert (ref.[6]).

$\delta^{13}C$ values of Toarcian and recent land plant constituents

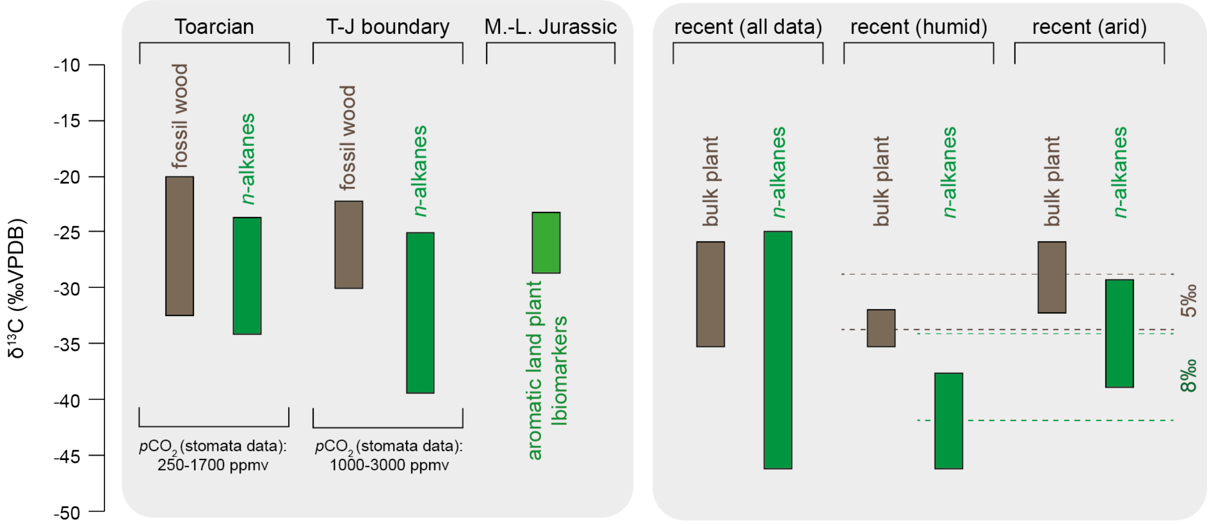

Fig. S4. Stable carbon isotope values of Toarcian and recent land plant constituents. Data are shown for the bulk organic matter of land plants (brown bars) and for long-chain *n*-alkanes (*n*C_{27} to *n*C_{35}). Bulk wood data for the Toarcian are form McElwain et al. (ref.[7]), Hesselbo et al. (ref.[8]), Hesselbo & Pienkowski (ref.[9]), Pienkowski et al. (ref.[10]). Compound specific $\delta^{13}C$ data are from French et al. (ref.[11]), Xu et al. (ref.[12]) and this study. $\delta^{13}C$ data of recent bulk plant material and *n*-alkanes (C3 plants only) are from Collister et al., (ref.[13]), Bi et al. (ref.[14]), Chikaraishi & Naraoka (ref.[15]), Garcin et al. (ref.[16]), Badewien et al. (ref.[17]) and Schwab et al. (ref.[18]). C3 plant growing under more arid conditions show a stronger enrichment in ^{13}C in both the bulk organic matter and the *n*-alkanes[18]. $\delta^{13}C_{wood}$ and $\delta^{13}C_{n\text{-alkane}}$ data from the Triassic-Jurassic (T-J) boundary are more enriched in ^{12}C when compared with data from the early Toarcian[19,20], which can be explained by higher pCO_2 levels during the T-J event (pCO_2 data are from Steinthorsdottir et al., (ref.[21])) then during the early Toarcian (pCO_2 data are from McElwain et al. (ref.[7]. Cooler climates and lower pCO_2 levels during the late Middle to Late Jurassic (see Dera et al., (ref.[22])) might also be reflected by the stronger ^{12}C depletion of aromatic land plant markers[23].

References

[1]Schubert, B.A., Jahren, A.H., 2013. Reconciliation of marine and terrestrial carbon isotope excursions based on changing atmospheric CO_2 levels. Nature Communications, DOI: 10.1038/ncomms2659.

[2]Beerling, D.J., Brentnall, S.J., 2007. Numerical evaluation of mechanisms driving Early Jurassic changes in global carbon cycling. Geology, v. 36, p. 231-234.

[3]Beerling, D.J., Lomas, M.R., Gröcke, D.R., 2002. On the nature of methane gas hydrate dissociation during the Toarcian and Aptian oceanic anoxic events: American Journal of Science, v. 302, p. 28–49, doi: 10.2475/ ajs.302.1.28.

[4]Anthony, K.M.W., Anthony, P., Grosse, G., Chanton, J., 2012. Geologic methane seeps along boundaries of Arctic permafrost thaw and melting glaciers. Nature Geoscience, v. 5, p. 419–426.

[5]Fischer, R.E., et al., 2017. Measurement of the ^{13}C isotopic signature of methane emissions from northern European wetlands. Global Biogeochemical Cycles, v. 31, p. 605-623.

[6]Cui, Y, Schubert, B.A., 2016. Quantifying uncertainty of past pCO2 determined from changes in C3 plant carbon isotope fractionation. Geochimica et Cosmochimica Acta 172, 127-138.

[7]McElwain, J.C., Wade-Murphy, J., Hesselbo, S.P., 2005. Changes in carbon dioxide during an oceanic anoxic event linked to intrusion into Gondwana coals. Nature, v. 435, p. 479–482.

[8]Hesselbo, S.P., Jenkyns, H.C., Duarte, L.V., Oliveira, L.C.V., 2007. Carbon-isotope record of the Early Jurassic (Toarcian) Oceanic Anoxic Event from fossil wood and marine carbonate (Lusitanian Basin, Portugal). Earth and Planetary Science Letters, v. 253, p. 455–470.

[9]Hesselbo, S.P., Pieńkowski, G, 2011. Stepwise atmospheric carbon-isotope excursion during the Toarcian Oceanic Anoxic Event (Early Jurassic, Polish Basin). Earth and Planetary Science Letters 301, 365–372.

[10]Pienkowski, G., Hodbod, M., Ullmann, C.V., 2016. Fungal decomposition of terrestrial organic matter accelerated Early Jurassic climate warming. Scientific Reports, 6, 31930, doi: 10.1038/srep31930.

[11]French, K.L., Sepulveda, J., Trabucho-Alexandre, J., Gröcke, D.R., Summons, R.E., 2014. Organic geochemistry of the early Toarcian oceanic anoxic event in Hawsker Bottoms, Yorkshire, England. Earth and Planetary Science Letters, v. 390, p. 116–127.

[12]Xu, W., Ruhl, M., Jenkyns, H.C., Hesselbo, S.P., Riding, J.B., Selby, D., Naafs, B.D.A., Weijers, J.W.H., Pancost, R.D., Tegelaar, E.W., Idiz, E.F., 2017. Carbon sequestration in an expanded lake system during the Toarcian oceanic anoxic event. Nature Geoscience, v. 10, p. 1–7.

[13]Collister, J.W., Rieley, G., Stern, B., Eglington, G., Fry, B., 1994. Compound-specific $\delta^{13}C$ analysis of leaf lipids from plants with differing carbon dioxide metabolism. Organic Geochemistry 21, 619-627.

[14]Bi, X., Sheng, G., Lui, X., Li, C., Fu, J., 2005. Molecular and carbon and hydrogen isotopic composition of *n*-alkanes in plant leaf waxes. Organic Geochemistry 36, 1405-1417.

[15]Chikaraishi, Y., Naraoka, H., 2007. $\delta^{13}C$ and δD relationships among three *n*-alkyl compound classes (*n*-alkanoic acid, *n*-alkane and *n*-alkanol) of terrestrial higher plants. Organic Geochemistry 38, 198215.

[16]Garcin, Y., Schefuß, E., Schwab, V.F., Garreta, V., Gleixner, G., Vincens, A., Todou, G., Séné, O., Onana, J.M., Achoundong, G., Sachse, D., 2014. Reconstructing C_3 and C_4 vegetation cover using *n*-alkane carbon isotope ratios in recent lake sediments from Cameroon, Western Central Africa. Geochimica et Cosmochimica Acta 142, 482–500.

[17]Badewien, T., Vogts, A., Rullkötter, J., 2015. n-Alkane distribution and carbon stable isotope composition in leaf waxes of C3 and C4 plants from Angola. Organic Geochemistry 89-90, 71-79.

[18]Schwab, V.F., Garcin, Y., Sachse, D., Todou, G., Séné, O., Onana, J.M., Achoundong, G., Gleixner, G., 2015. Effect of aridity on $\delta^{13}C$ and δD values of C_3 plant- and C_4 graminoid-derived leaf wax lipids from soils along an environmental gradient in Cameroon (Western Central Africa). Organic Geochemistry78, 99-109.

[19]Whiteside, J.H., Olsen, P.E., Eglinton, T., Brookfield, M.E., Sambrotto, R.N., 2010. Compound-specific carbon isotopes from Earth's largest flood basalt eruptions directly linked to the end-Triassic mass extinction. PNAS, v. 107, p. 6721-6725.

[20]Ruhl, M., Boni, N.R., Reichert, G.J., Sinninghe Damsté, J.S., Kürschner, W.M., 2011. Atmospheric Carbon Injection Linked to End-Triassic Mass Extinction. Science 333, 430-43.

[21]Steinthorsdottir, M., Jeram, A.J., McElwain, J.C., 2011. Extremely elevated CO_2 concentrations at the Triassic/Jurassic boundary. Palaeogeography, Palaeoclimatology, Palaeoecology 308, 418 – 432.

[22]Dera, G., Brigaud, B., Monna, F., Laffot, R., Pucéat, E., Deconinck, J.-F., Pellenard, P., Joachimski, M.M., Durlet, C., 2011. Climate ups and downs in a disturbed Jurassic world. Geology, v. 39, p. 215–218.

[23]Grice, K., Riding, J.B., Foster, C.B., Naeher, S., Greenwood, P.F., 2015. Vascular plant biomarker distributions and stable carbon isotopic signatures from the Middle and Upper Jurassic (Callovian–Kimmeridgian) strata of Staffin Bay, Isle of Skye, northwest Scotland. Palaeogeography, Palaeoclimatology, Palaeoecology, v. 440, p. 307–315.
